# Molecular mechanism underlying miR-204-5p regulation of adipose-derived stem cells differentiation into cells from three germ layers

**DOI:** 10.1038/s41420-024-01852-4

**Published:** 2024-02-22

**Authors:** Zhimin Wang, Meiyu Bi, Xiaoshu Zhe, Xiao Wang, Bai Dai, Xiaoyu Han, Bingxu Ren, Hao Liang, Dongjun Liu

**Affiliations:** 1 State Key Laboratory of Reproductive Regulation and Breeding of Grassland Livestock, School of Life Sciences, Inner Mongolia University, Hohhot, 010070 P.R. China; 2 Reproductive Medicine Center, Affiliated Hospital of Inner Mongolia Medical University, Hohhot, 010050 P.R. China

**Keywords:** Mesenchymal stem cells, Cell biology, Stem-cell differentiation, Differentiation, Epigenetics

## Abstract

The limited differentiation ability of adipose-derived stem cells (ADSCs) limits their application in stem cell therapy and regenerative medicine. Here, we explore the molecular mechanism by which miR-204-5p regulates ADSCs differentiation into cells derived from the three germ layers (i.e., adipocytes, neurocytes, and hepatocytes). Although miR-204-5p overexpression inhibited ADSCs differentiation into adipocytes, neurocyte and hepatocyte differentiation were promoted. Mechanistically, miR-204-5p inhibited the expression of PPARG by regulating the AMPK signaling pathway, thereby inhibiting ADSCs differentiation into adipocytes. Further, miR-204-5p regulated JAG1/NOTCH3 axis for the inhibition of differentiation into adipocytes and promotion of differentiation into neurocytes. miR-204-5p might also promote ADSCs differentiation into hepatocytes by upregulating E2F8. The findings of this study provide novel insights into the regulatory mechanisms underlying early embryonic development and will help to facilitate the application of ADSCs in stem cell therapy and regenerative medicine.

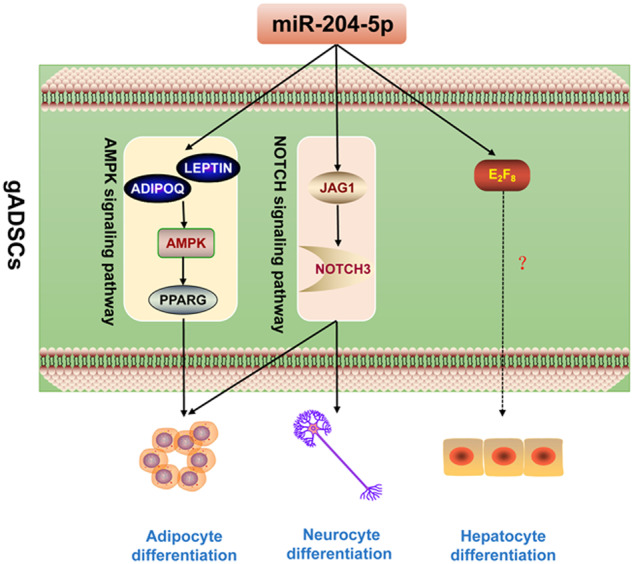

## Introduction

The application of cell therapy in medicine has attracted attention; stem cells represent a core cell therapy element. Diabetes is a chronic disease that seriously threatens the health of patients and lowers their quality of life, for which a cure remains lacking. Stem cell therapy is a potential treatment strategy against Type I diabetes [1]. Furthermore, stem cell therapy provides a new approach to the treatment of neurodegenerative diseases [2]. Stem cells can differentiate into cells in the inner, middle, and outer germ layers under certain conditions and simulate embryonic development in vitro, forming various types of cells, tissues, and organs [3–5]. Embryonic stem cells (ESCs) and induced pluripotent stem cells (iPSCs) have potential application in cell therapy and regenerative medicine owing to their differentiation potential; however, challenges associated with ethical issues, genomic instability, tumorigenicity, and immune rejection, remain to be addressed [6, 7]. Therefore, mesenchymal stem cells (MSCs) from different sources have been widely explored as replacements for ESCs and iPSCs in stem cell therapy and regenerative medicine [8]. Adipose-derived stem cells (ADSCs) have several advantages, such as easy access, abundant sources, stable cell traits, safety and reliability, and strong immunosuppressive properties [9, 10]. ADSCs have self-renewal and multi-directional differentiation potential and can differentiate into multiple cell types of the three germ layers and subsequent tissues and organs [11–14]. Therefore, ADSCs are ideal seed cells for stem cell therapy and regenerative medicine and provide a cell model for studying early embryonic development.

Despite these advantages, ADSCs have been reported to only differentiate into a limited number of specific cell types, thereby limiting their clinical application [15]. The multi-directional differentiation process of ADSCs is regulated by multiple factors such as gene expression, signaling pathways, epigenetics, and extracellular signaling [12–14, 16], in which microRNAs (miRNAs) play an important role as a component of epigenetic modification [17]. miRNAs are small non-coding RNAs that regulate gene expression [18] and play an important regulatory role in ADSCs differentiation. miR-26a regulates ADSCs differentiation into adipocytes through the CDK5/FOXC2 pathway [19], while miR-130a-3p regulates ADSCs differentiation into osteoblasts by acting on SIRT7/wnt/β-Catenin axis [20]. miRNA-124 can also regulate ADSCs differentiation into neurocytes by targeting Sp1 mRNA [21]. miR-21-5p inhibits the Notch signaling pathway and regulates ADSCs differentiation into Schwann cells by targeting Rbpj [22], while miR-122 overexpression and let-7f silencing can significantly enhance the ability of ADSCs to differentiate into hepatocytes [23]. These findings illustrate that regulating the expression of miRNAs can improve the ability of ADSCs to differentiate into specific cells of the three germ layers, thus allowing for broader applications of ADSCs in stem cell therapy and regenerative medicine.

miR-204-5p is highly expressed in the human fetal retinal pigment epithelium and zebrafish lens [24]. miR-204-5p is also expressed in different mammalian tissues, and its sequence is highly conserved, with similar secondary structure and mature sequence in different animals, suggesting its involvement in similar biological and developmental processes in different animals [25]. Recent evidence suggests that miR-204-5p is a tumor suppressor gene involved in regulating tumor cell proliferation, stem cell characteristics, metastasis, apoptosis, chemotherapy resistance, and autophagy [26]. Additionally, miR-204-5p plays an important regulatory role in ADSCs differentiation into mesodermal cells and promotes ADSCs differentiation into adipocytes by controlling the expression of DVL3, a key regulator of the Wnt/β-catenin signaling pathway [27]. Furthermore, the miR-204-5p/FOXC1/GDF7 axis regulates the osteogenic differentiation of ADSCs through the AKT and p38 signaling pathways [28]. However, whether miR-204-5p has regulatory effects on ADSCs differentiation from the three germ layers into other cell types and the associated molecular mechanisms remain nebulous.

As an effective treatment strategy in regenerative medicine, stem cell therapy must be thoroughly verified in animal models before clinical trials. Previous studies have shown that the plasticity, differentiation potential, pluripotency, and immune regulation characteristics of livestock ADSCs are consistent with those of human ADSCs [29, 30]; therefore, using ADSCs from large animal models as research objects will help to determine the effectiveness and safety of cell therapy in humans [31]. In this study, goat ADSCs (gADSCs) cell models were used to explore the molecular mechanism by which miR-204-5p regulates ADSCs differentiation into cells from the three germ layers (adipocytes, neurocytes, and hepatocytes). The results of this study will help to facilitate the accurate induction and control of ADSCs differentiation into different cell types using miRNAs. This study also provides novel insights into the regulatory mechanism of early embryonic development and will help to facilitate the establishment of a directional differentiation system of ADSCs for stem cell therapy and regenerative medicine.

## Results

### Detection of infection efficiency of miR-204-5p up lentiviral vector

Uninfected gADSCs were used for the Control group; gADSCs infected with the empty lentiviral vector were labeled as the LV_Con group, and gADSCs infected with the miR-204-5p lentiviral vector were labeled as the LV_miR group. Fluorescence microscopy showed no green fluorescence in the Control group, whereas a large amount of green fluorescence was observed in both the LV_Con and LV_miR groups (Fig. S1A). Flow cytometric analysis showed that cells expressing the green fluorescent protein in the LV_Con and LV_miR groups accounted for > 97% of selected cells (Fig. S1B). Real-time quantitative polymerase chain reaction (qPCR) revealed that miR-204-5p was highly expressed in the LV_miR group (Fig. S1C). These results indicate that lentiviral vectors have high infection efficiency and miR-204-5p can be stably overexpressed in gADSCs.

### Effects of overexpression of miR-204-5p on the differentiation of gADSCs into adipocytes

**Fig. 1:**
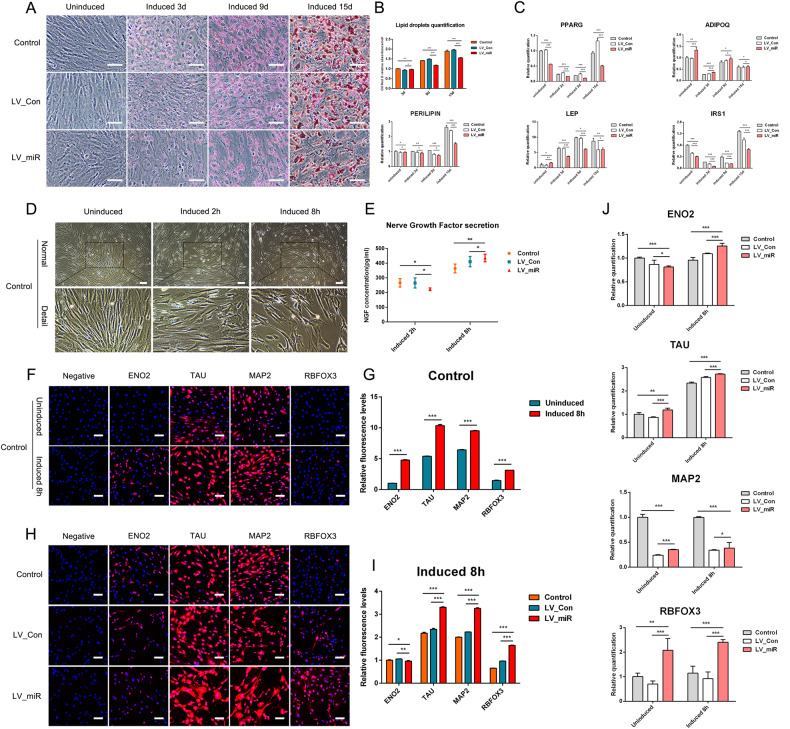
Effects of overexpression of miR-204-5p on gADSCs differentiation into adipocytes and neurocytes. **A** Identification of adipocytes using oil red O staining (Bar: 100 μm). **B** Determination of lipid droplet content. **C** Transcription of adipocyte marker genes PPARG, ADIPOQ, PERILIPIN, LEP, and IRS1 detected using real-time qPCR. **D** Cell morphology during differentiation of Control group into neurocytes (Bar: 100 μm). **E** NGF in the supernatant of neurocytes detected using ELISA. **F** Cellular immunofluorescence showing the expression of neurocyte marker genes ENO2, TAU, MAP2, and RBFOX3 before and after differentiation in the Control group (Bar: 100 μm). **G** Fluorescence intensity analysis of **F**. **H** Cellular immunofluorescence showing the expression of ENO2, TAU, MAP2, and RBFOX3 after differentiation in the Control, LV_Con, and LV_miR groups (Bar: 100 μm). **I** Fluorescence intensity analysis of **H**. **J** Transcription of ENO2, TAU, MAP2, and RBFOX3 detected using real-time qPCR. Data were presented as mean ± SD; P values were determined by a two-tailed unpaired t test, *P > 0.05, **0.01 < *P* < 0.05, ***P < 0.01.

Differentiation in the Control, LV_Con, and LV_miR groups into adipocytes was simultaneously induced. Oil Red O staining and lipid droplet content analysis showed that the volume and number of lipid droplets increased significantly with the induction time. In addition, compared with those in the Control and LV_Con groups, the number and content of lipid droplets in the LV_miR group were lower, and the volume was slightly smaller (Fig. 1A, B).

Transcription of the adipocyte marker genes peroxisome proliferator-activated receptor gamma (PPARG), adiponectin (ADIPOQ), PERILIPIN, leptin (LEP), and insulin receptor substrate 1 (IRS1) during differentiation was detected using real-time qPCR (Fig. 1C). Compared with their expression in the Control and LV_Con groups, PPARG and IRS1 were downregulated in the LV_miR group during differentiation. ADIPOQ and LEP were highly transcribed when not induced. With longer induction, although ADIPOQ remained highly transcribed, the gap gradually narrowed, and LEP showed a varying degree of decrease. The transcription level of PERILIPIN showed no statistically significant differences during the initial induction but significantly decreased on day 15.

These results indicate that gADSCs can differentiate into adipocytes in vitro and that overexpression of miR-204-5p has inhibitory effects on the differentiation of gADSCs into adipocytes.

### Effects of overexpression of miR-204-5p on the differentiation of gADSCs into neurocytes

Differentiation of Control, LV_Con, and LV_miR groups into neurocytes was simultaneously induced. Morphological observations showed that after 2 h of induction, cell morphology began to change, cells retracted, and part of the cytoplasm extended outward. After 8 h of induction, cell protrusions were more obvious, cells were interlaced and connected to a network, and the morphology of the neurocytes was obvious (Fig. 1D). Detection of nerve growth factor (NGF) content showed that NGF secreted by the LV_miR group was higher than that in the Control and LV_Con groups after 8 h (Fig. 1E).

Cellular immunofluorescence was used to identify the expression of the neurocyte marker genes enolase 2 (ENO2), tubulin-associated unit (TAU), microtubule-associated protein 2 (MAP2), and RNA binding protein Fox-1 homolog 3 (RBFOX3) before and after differentiation. The fluorescence intensities of ENO2, TAU, MAP2, and RBFOX3 were significantly higher in cells induced for 8 h than in uninduced cells (Fig. 1F, G). In addition, the expression of TAU, MAP2, and RBFOX3 was significantly higher in the LV_miR group than in the Control and LV_Con groups after differentiation (Fig. 1H, I).

Transcription of the neurocyte marker genes ENO2, TAU, MAP2, and RBFOX3 before and after differentiation was detected using real-time qPCR (Fig. 1J). The transcription levels of ENO2, TAU, and RBFOX3 were higher in cells induced for 8 h than in uninduced cells; however, MAP2 expression did not show this pattern. In addition, compared with their expression in the Control and LV_Con groups, ENO2, TAU, and RBFOX3 were upregulated in the LV_miR group after 8 h, and the transcription level of MAP2 was higher than that in the LV_Con group.

These results indicate that gADSCs can differentiate into neurocytes in vitro and the overexpression of miR-204-5p can promote the differentiation of gADSCs into neurocytes.

### Effects of overexpression of miR-204-5p on the differentiation of gADSCs into hepatocytes

**Fig. 2:**
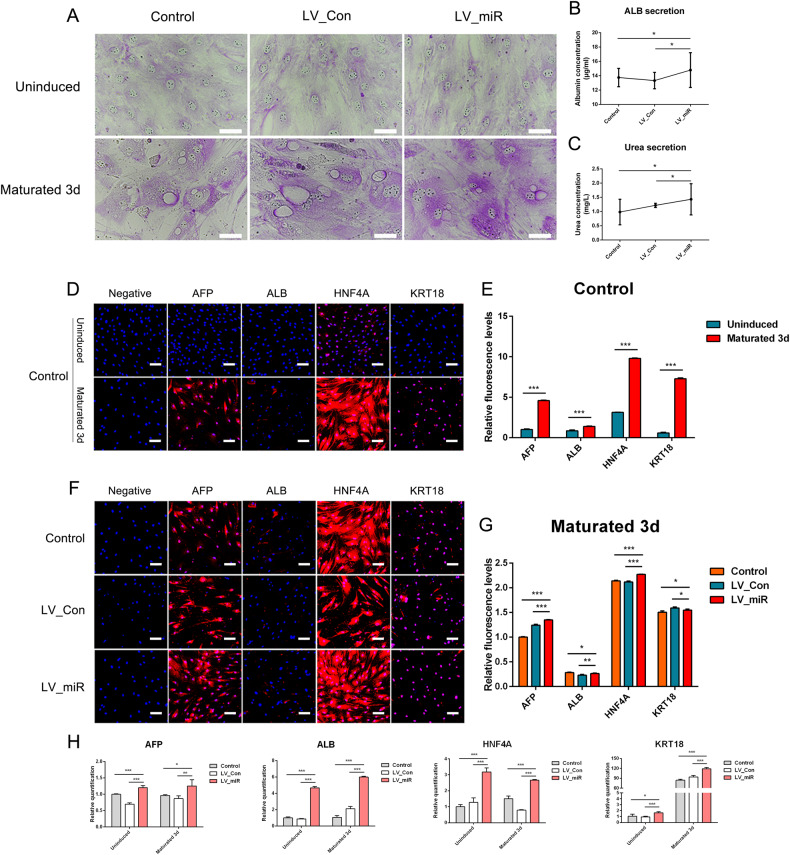
Effects of overexpression of miR-204-5p on gADSCs differentiation into hepatocytes. **A** Glycogen vesicles in hepatocytes detected using glycogen PAS staining (Bar: 50 μm). **B** ALB in the supernatant of hepatocytes detected using ELISA. **C** Determination of urea in the supernatant of hepatocytes. **D** Cellular immunofluorescence identified the expression of hepatocyte marker genes AFP, ALB, HNF4A, and KRT18 before and after differentiation in the Control group (Bar: 100 μm). **E** Fluorescence intensity analysis of **D**. **F** Cellular immunofluorescence identified the expression of AFP, ALB, HNF4A, and KRT18 after differentiation in the Control, LV_Con, and LV_miR groups (Bar: 100 μm). **G** Fluorescence intensity analysis of **F**. **H** Transcription of AFP, ALB, HNF4A, and KRT18 detected using real-time qPCR. Data were presented as mean ± SD; *P* values were determined by a two-tailed unpaired t test, *P > 0.05, **0.01 < *P* < 0.05, ***P < 0.01.

Differentiation of Control, LV_Con, and LV_miR groups into hepatocytes was simultaneously induced. The results of glycogen PAS staining showed that the differentiated hepatocytes had varying shades of purple-red within the cells after 3 days of maturation. The nucleus was visible and tightly surrounded by a large number of vesicular structures (i.e., glycogen vesicles) (Fig. 2A). The detection of albumin (ALB) and urea content showed that ALB and urea secreted by the LV_miR group were higher than that of the Control and LV_Con groups following differentiation (Fig. 2B, C).

Cellular immunofluorescence was used to identify the expression of hepatocyte marker genes alpha fetoprotein (AFP), ALB, hepatocyte nuclear factor 4-alpha (HNF4A), and keratin 18 (KRT18) before and after differentiation. The fluorescence intensities of AFP, ALB, HNF4A, and KRT18 were significantly higher in cells that matured by day 3 than in uninduced cells (Fig. 2D, E). In addition, compared with those in the Control and LV_Con groups, no statistically significant difference was observed in the expression levels of ALB or KRT18 in the LV miR-204-5p group after differentiation induction, whereas the expression levels of AFP and HNF4A were significantly increased (Fig. 2F, G).

Transcription of the hepatocyte marker genes ENO2, TAU, MAP2, and RBFOX3 before and after differentiation was detected using real-time qPCR (Fig. 2H). Compared with those in the uninduced cells, the transcription levels of AFP, ALB, HNF4A, and KRT18 increased in cells after 3 days of maturation, wherein that of KRT18 was more obvious. In addition, AFP, ALB, HNF4A, and KRT18 were upregulated in the LV_miR group, compared with their expressions in the Control and LV_Con groups, after 3 days of maturation.

These results indicate that gADSCs can differentiate into hepatocytes in vitro and overexpression of miR-204-5p promotes the differentiation of gADSCs into hepatocytes.

### The molecular pathway of miR-204-5p regulating the differentiation of gADSCs into adipocytes

**Fig. 3:**
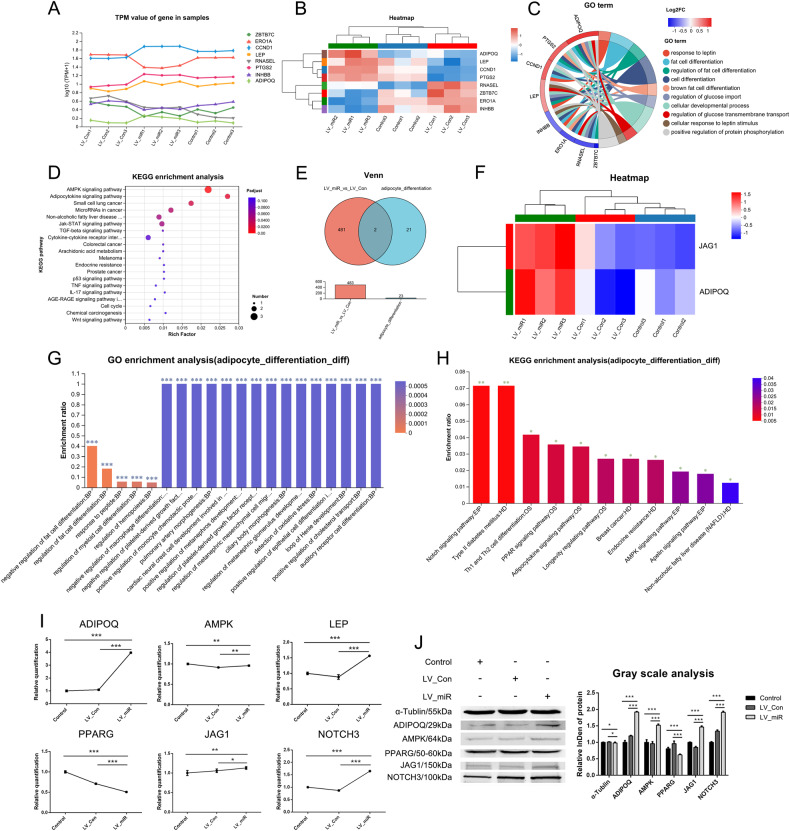
Analysis of differential genes and differential proteins associated with adipocyte differentiation. **A** Line chart of expression of differential genes associated with adipocyte differentiation. **B** Clustering heatmap of differential genes associated with adipocyte differentiation. **C** Chordal graph of GO enrichment of differential genes associated with adipocyte differentiation. **D** KEGG enrichment analysis of differential genes associated with adipocyte differentiation. **E** Venn analysis of the adipocyte_differentiation and LV_Con_vs_LV_miR groups. **F** Clustering heatmap of differential proteins associated with adipocyte differentiation. **G** GO enrichment analysis of differential proteins associated with adipocyte differentiation. **H** KEGG enrichment analysis of differential proteins associated with adipocyte differentiation. **I** Transcription of differential genes associated with adipocyte differentiation detected using real-time qPCR. **J** Expression of differential proteins associated with adipocyte differentiation detected using western blotting. Data were presented as mean ± SD; *P* values were determined by a two-tailed unpaired *t* test, *P > 0.05, **0.01 < *P* < 0.05, ***P < 0.01.

Transcriptome sequencing was performed on the Control, LV_Con, and LV_miR group cells. A total of 18,998 genes and 1,247 differential genes were obtained, of which the LV_Con_vs_LV_miR group had 255 differential genes. According to functional annotation, eight differentially expressed genes were associated with adipocyte differentiation in the LV_Con_vs_LV_miR group, namely ZBTB7C, ERO1A, CCND1, LEP, RNASEL, PTGS2, INHBB, and ADIPOQ. The corresponding line chart of the gene expression is shown in Fig. 3A, while the clustering heat map is in Fig. 3B. GO enrichment analysis revealed that these genes were involved in cell differentiation, cellular developmental process, and fat cell differentiation (Fig. 3C). KEGG enrichment analysis revealed that these genes were enriched in signaling pathways such as AMPK, Adipocytokine, and JAK-STAT (Fig. 3D).

Proteome sequencing identified 6,569 proteins and 1,610 differential proteins, among which the LV_Con_vs_LV_miR group had 483 differential proteins. We identified 23 out of 6,569 proteins with functional annotations associated with adipocyte differentiation, named the adipocyte_differentiation group. Venn analysis of the adipocyte_differentiation and LV_Con_vs_LV_miR group revealed that only ADIPOQ and JAG1 were associated with adipocyte differentiation in the LV_Con_vs_LV_miR group (Fig. 3E) and were upregulated (Fig. 3F). GO enrichment analysis revealed that these two proteins are involved in adipocyte differentiation and play a negative regulatory role in this process (Fig. 3G). KEGG enrichment analysis revealed that these two proteins were enriched in signaling pathways such as those of AMPK, NOTCH, and PPAR (Fig. 3H).

ADIPOQ was detected in the differential expression analysis of the transcriptome and proteome and negatively regulated adipocyte differentiation. Real-time qPCR and western blotting showed that ADIPOQ expression was upregulated in the LV_miR group (Fig. 3I, J). ADIPOQ was a key factor in activating the AMPK signaling pathway, and AMPK, the core protein of this pathway, was also upregulated in the LV_miR group. Two important proteins associated with adipocyte differentiation, LEP and PPARG, were also involved in this pathway. LEP is a key factor in activating AMPK, whereas PPARG is located downstream of AMPK. Real-time qPCR showed that LEP was upregulated and PPARG was downregulated in the LV_miR group; no statistically significant difference in AMPK expression was observed (Fig. 3I). Western blotting showed that AMPK was upregulated and PPARG was downregulated in the LV_miR group; however, LEP expression was not detected (Fig. 3J). JAG1 is a key protein that activates the NOTCH signaling pathway and negatively regulates adipocyte differentiation. Real-time qPCR and western blotting showed that JAG1 and its downstream target NOTCH3 were upregulated in the LV_miR group (Fig. 3I, J). These results indicate that miR-204-5p may inhibit the differentiation of gADSCs into adipocytes by acting on the AMPK signaling pathway and the JAG1/NOTCH3 axis.

### The molecular pathway of miR-204-5p regulating the differentiation of gADSCs into neurocytes

**Fig. 4:**
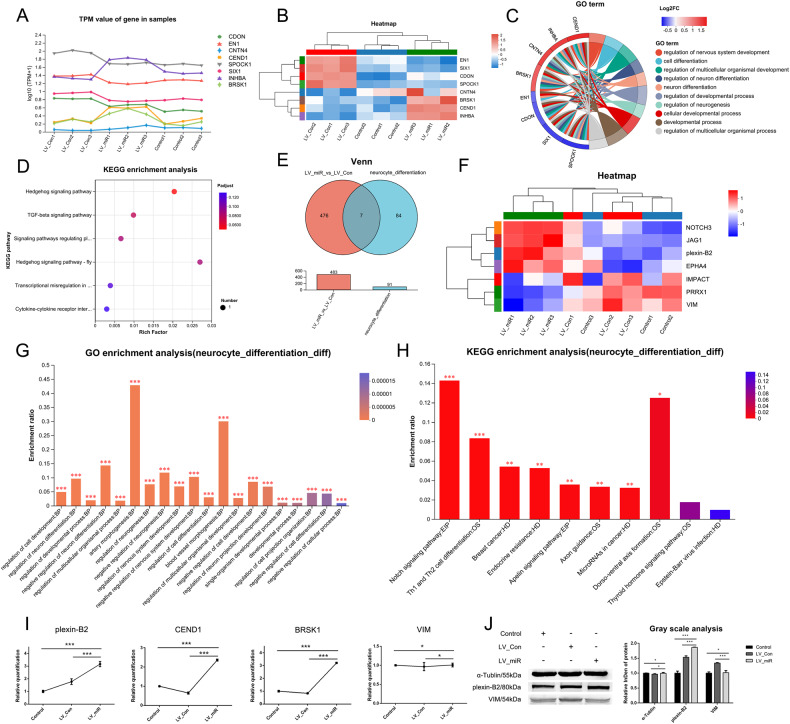
Analysis of differential genes and differential proteins associated with neurocyte differentiation. **A** Line chart of expression of differential genes associated with neurocyte differentiation. **B** Clustering heatmap of differential genes associated with neurocyte differentiation. **C** Chordal graph of GO enrichment of differential genes associated with neurocyte differentiation. **D** KEGG enrichment analysis of differential genes associated with neurocyte differentiation. **E** Venn analysis of the neurocyte_differentiation and LV_Con_vs_LV_miR groups. **F** Clustering heatmap of differential proteins associated with neurocyte differentiation. **G** GO enrichment analysis of differential proteins associated with neurocyte differentiation. **H** KEGG enrichment analysis of differential proteins associated with neurocyte differentiation. **I** Transcription of differential genes associated with neurocyte differentiation detected using real-time qPCR. **J** Expression of differential proteins associated with neurocyte differentiation detected using western blotting. Data were presented as mean ± SD; *P* values were determined by a two-tailed unpaired t test, *P > 0.05, **0.01 < *P* < 0.05, ***P < 0.01.

Functional annotation of the transcriptome showed that eight differentially expressed genes were associated with neurocyte differentiation in the LV_Con_vs_LV_miR group, namely CDON, EN1, CNTN4, CEND1, SPOCK1, SIX1, INHBA, and BRSK1. The corresponding line chart of gene expression is shown in Fig. 4A, while the clustering heat map in Fig. 4B. GO enrichment analysis revealed that these genes are involved in the regulation of nervous system development, neuron differentiation, and neurogenesis (Fig. 4C), while KEGG enrichment analysis showed that these genes were enriched in signaling pathways such as Hedgehog and TGF-beta (Fig. 4D).

According to the functional annotation of the proteome, 91 proteins were associated with neurocyte differentiation, and these were named as the neurocyte_differentiation group. Venn analysis of the neurocyte_differentiation and LV_Con_vs_LV_miR groups revealed that seven differentially expressed proteins were associated with neurocyte differentiation in the LV_Con_vs_LV_miR group (Fig. 4E), namely NOTCH3, IMPACT, PRRX1, JAG1, EPHA4, VIM, and plexin-B2, of which four were upregulated and three were downregulated (Fig. 4F). GO enrichment analysis revealed that these proteins were mainly involved in the regulation of cell differentiation, neuron differentiation, and neurogenesis (Fig. 4G), while KEGG enrichment analysis revealed that these proteins were enriched in signaling pathways, such as NOTCH, Apelin, and Axon guidance (Fig. 4H).

Among the differential proteins identified using proteome analysis, JAG1 and NOTCH3, two key proteins in the NOTCH signaling pathway, have important regulatory effects on neurocyte differentiation and were upregulated in the LV_miR group (Fig. 3I, J). In addition, the differentially expressed proteins plexin-B2 and VIM, and genes CEND1 and BRSK1 also play important roles in neurocyte differentiation. Among these, plexin-B2, CEND1, and BRSK1 play positive regulatory roles in neurocyte differentiation, whereas VIM plays a negative regulatory role. Real-time qPCR showed that plexin-B2, CEND1, and BRSK1 were upregulated in the LV_miR group; however, there was no statistically significant difference in VIM (Fig. 4I). Western blotting showed that plexin-B2 was upregulated in the LV_miR group and the expression of VIM was significantly lower than that in the LV_Con group. In addition, CEND1 and BRSK1 expression was not detected (Fig. 4J). These results indicate that the promoting effect of miR-204-5p on the differentiation of gADSCs into neurocytes may be achieved by acting on the JAG1/NOTCH3 axis. In addition, miR-204-5p may promote the differentiation of gADSCs into neurocytes by upregulating the expression of plexin-B2, CEND1, and BRSK1 and downregulating that of VIM.

### The molecular pathway of miR-204-5p regulating the differentiation of gADSCs into hepatocytes

**Fig. 5:**
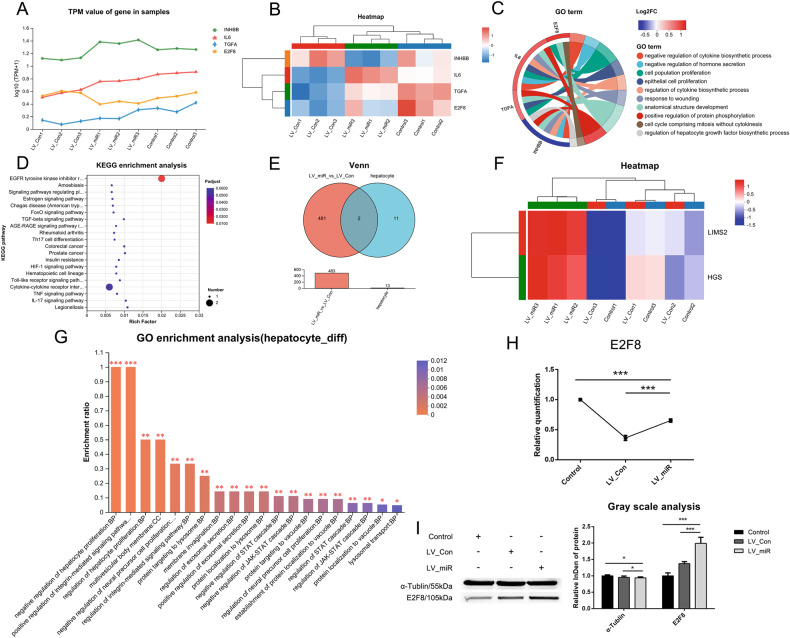
Analysis of differential genes and differential proteins associated with hepatocytes. **A** Line chart of expression of differential genes associated with hepatocytes. **B** Clustering heatmap of differential genes associated with hepatocytes. **C** Chordal graph of GO enrichment of differential genes associated with hepatocytes. **D** KEGG enrichment analysis of differential genes associated with hepatocytes. **E** Venn analysis of the hepatocyte and LV_Con_vs_LV_miR groups. **F** Clustering heatmap of differential proteins associated with hepatocytes. **G** GO enrichment analysis of differential proteins associated with hepatocytes. **H** Transcription of differential genes associated with hepatocyte differentiation detected using real-time qPCR. **I** Expression of differential proteins associated with hepatocyte differentiation detected using western blotting. Data were presented as mean ± SD; *P* values were determined by a two-tailed unpaired *t* test, *P > 0.05, **0.01 < *P* < 0.05, ***P < 0.01.

The functional annotation of the transcriptome showed that four differentially expressed genes were associated with hepatocytes in the LV_Con_vs_LV_miR group, namely IL6, TGFA, E2F8, and INHBB. The corresponding line chart of gene expression is shown in Fig. 5A, while the clustering heat map in Fig. 5B. GO enrichment analysis revealed that these genes are involved in the regulation of cytokine and hepatocyte growth factor biosynthetic process (Fig. 5C), while KEGG enrichment analysis revealed that these genes were mainly enriched in signaling pathways such as FoxO, TGF-beta, and HIF-1 (Fig. 5D).

Functional annotation of the proteome identified 13 proteins associated with hepatocytes, named the hepatocyte group. Venn analysis of hepatocyte and LV_Con_vs_LV_miR groups revealed that only two differentially expressed proteins were associated with hepatocytes in the LV_Con_vs_LV_miR group (Fig. 5E) and upregulated in the LV_miR group (Fig. 5F). GO enrichment analysis revealed that the two differentially expressed proteins were involved in the regulation of hepatocyte proliferation and integrin-mediated signaling pathway (Fig. 5G), none of which were associated with hepatocyte differentiation.

Only E2F8 was associated with hepatocyte differentiation. Real-time qPCR showed that E2F8 was upregulated in the LV_miR group compared with the LV_Con group (Fig. 5H). Western blotting showed that the expression of E2F8 in the LV_miR group was significantly higher than in the Control and LV_Con groups (Fig. 5I). These results indicate that the promoting effect of miR-204-5p on the differentiation of gADSCs into hepatocytes may be achieved by increasing the expression of E2F8.

### The effects of AMPK and NOTCH signaling pathways on the differentiation of gADSCs into adipocytes

**Fig. 6:**
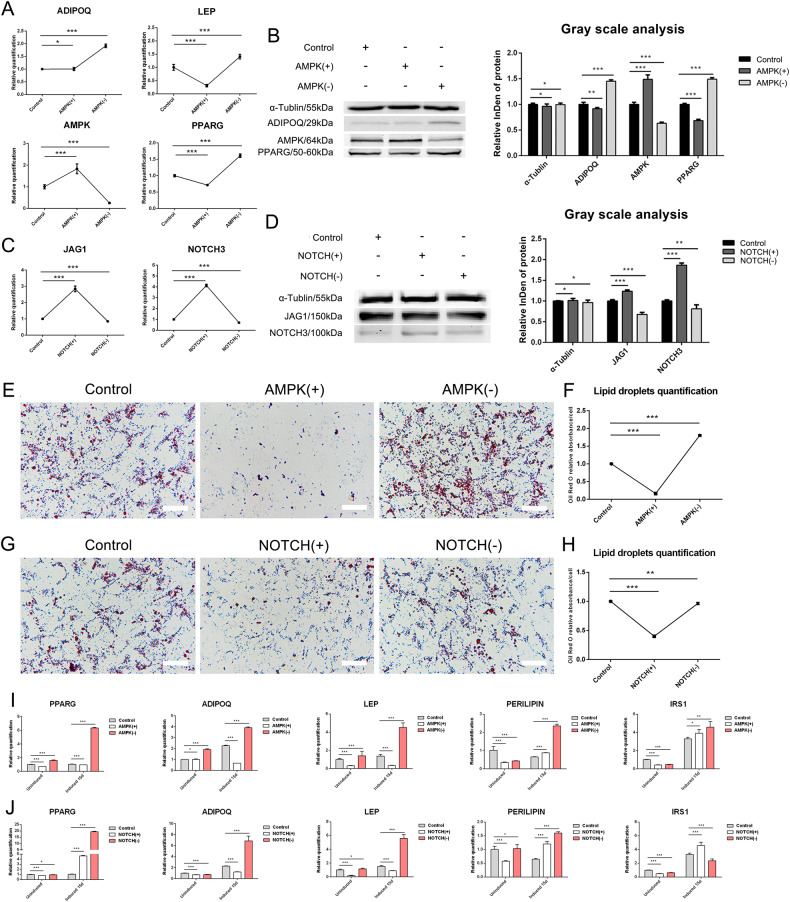
Effects of AMPK and NOTCH signaling pathways on gADSCs differentiation into adipocytes. **A**, **B** Expression of key genes in the AMPK signaling pathway detected using real-time qPCR and western blotting. **C**, **D** Expression of key genes in the NOTCH signaling pathway detected using real-time qPCR and western blotting. **E**, **F** Effects of AMPK signaling pathway on gADSCs differentiation into adipocytes detected using oil red O staining and quantitative detection of lipid droplets (Bar: 50 μm). **G**, **H** Effects of NOTCH signaling pathway on gADSCs differentiation into adipocytes detected using oil red O staining and quantitative detection of lipid droplets (Bar: 50 μm). **I** Transcription of adipocyte marker genes before and after differentiation in AMPK(+) and AMPK(−) groups detected using real-time qPCR. **J** Transcription of adipocyte marker genes before and after differentiation in NOTCH(+) and NOTCH(−) groups detected using real-time qPCR. Data were presented as mean ± SD; *P* values were determined by a two-tailed unpaired *t* test, *P > 0.05, **0.01 < *P* < 0.05, ***P < 0.01.

The gADSCs were treated with the AMPK activator (AMPK(+)) AICAR and inhibitor (AMPK(−)) dorsomorphin (Compound C) 2HCl and the NOTCH activator (NOTCH(+)) valproic acid (VPA) and inhibitor (NOTCH(−)) LY411575, respectively, using untreated gADSCs as control. The expression of key genes in the AMPK and NOTCH signaling pathways was analyzed using real-time qPCR and western blotting. Real-time qPCR showed that, compared with their levels in the control group, AMPK was upregulated and LEP and PPARG were downregulated in the AMPK(+) group, whereas no statistically significant difference in ADIPOQ expression was observed. In the AMPK(−) group, ADIPOQ, LEP, and PPARG were upregulated, whereas AMPK was downregulated (Fig. 6A). Western blotting showed that AMPK was upregulated, ADIPOQ and PPARG were downregulated, and no expression of LEP was detected in the AMPK (+) group. The results of the AMPK(−) group were opposite to those of the AMPK(+) group (Fig. 6B). In addition, real-time qPCR and western blotting showed that compared with their levels in the control group, JAG1 and NOTCH3 were upregulated in the NOTCH(+) group but downregulated in the NOTCH(−) group (Fig. 6C, D).

Oil red O staining and quantitative detection of lipid droplets were performed on adipocytes differentiated from the Control, AMPK(+), AMPK(−), NOTCH(+), and NOTCH(−) groups. Compared with those in the Control group, the number and content of lipid droplets were lower in the AMPK(+) group but higher in the AMPK(−) group (Fig. 6E, F). In addition, the number and content of lipid droplets in the NOTCH(+) group were lower than those in the Control group; no statistically significant difference between the NOTCH(−) and Control groups was observed (Fig. 6G, H). Real-time qPCR showed that after differentiation, compared with their levels in the Control group, PPARG, ADIPOQ, and LEP were downregulated, and PERILIPIN and IRS1 were upregulated in the AMPK(+) group; however, their transcription levels were lower than those in the AMPK(−) group. PPARG, ADIPOQ, LEP, PERILIPIN, and IRS1 were upregulated in the AMPK(−) group (Fig. 6I). In addition, ADIPOQ and LEP were downregulated, while PPARG, PERILIPIN, and IRS1 were upregulated in the NOTCH(+) group. In the NOTCH(−) group, PPARG, ADIPOQ, LEP, and PERILIPIN were upregulated, whereas only IRS1 was downregulated (Fig. 6J). Therefore, the activation of the AMPK signaling pathway inhibited gADSCs differentiation into adipocytes, whereas inhibition of the AMPK signaling pathway had the opposite effect. In addition, activation of the NOTCH signaling pathway inhibited the differentiation of gADSCs into adipocytes, but the promotional effect of inhibiting this pathway on the differentiation of gADSCs into adipocytes was not obvious.

### The effects of NOTCH signaling pathways on the differentiation of gADSCs into neurocytes

The gADSCs were treated with the NOTCH activator (NOTCH(+)) valproic acid (VPA) and inhibitor (NOTCH(−)) LY411575, respectively, using untreated gADSCs as a control. The expression of key genes in the NOTCH signaling pathway was detected using real-time qPCR and western blotting. JAG1 and NOTCH3 were upregulated in the NOTCH(+) group, while downregulated in the NOTCH(−) group (Fig. 6C, D).

**Fig. 7:**
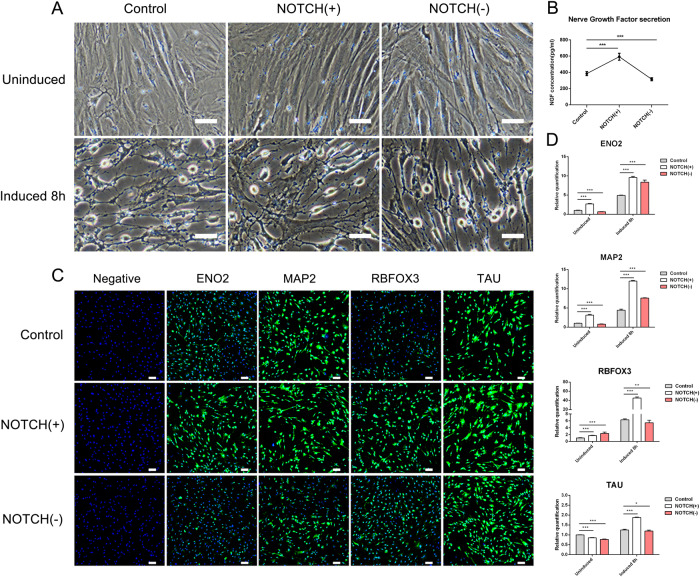
Effects of NOTCH signaling pathway on gADSCs differentiation into neurocytes. **A** Cell morphology before and after differentiation of the Control, NOTCH(+), and NOTCH(−) groups into neurocytes (Bar: 50 μm). **B** NGF in the supernatant of neurocytes detected using ELISA. **C** Cellular immunofluorescence identified the expression of ENO2, TAU, MAP2, and RBFOX3 after differentiation in the Control, NOTCH(+), and NOTCH(−) groups (Bar: 100 μm). **D** Transcription of ENO2, TAU, MAP2, and RBFOX3 before and after differentiation in the Control, NOTCH(+) and NOTCH(−) groups detected using real-time qPCR. Data were presented as mean ± SD; *P* values were determined by a two-tailed unpaired *t* test, *P > 0.05, **0.01 < *P* < 0.05, ***P < 0.01.

Differentiation of the Control, NOTCH (+), and NOTCH(−) groups into neurocytes was simultaneously induced. Morphological observations showed that following an 8 h induction, the cell body retracted, part of the cytoplasm extended outward, the cells were interconnected into a network, and the morphology of the neurocytes was evident (Fig. 7A). NGF content was higher in the NOTCH (+) group, but lower in the NOTCH(−) group, than in the Control group (Fig. 7B). Cellular immunofluorescence showed that the fluorescence intensity of ENO2, MAP2, RBFOX3, and TAU in the NOTCH(+) group was higher than the corresponding intensities in the Control group, whereas they were lower in the NOTCH (−) group, except for RBFOX3 (Fig. 7C). Real-time qPCR showed that following an 8 h induction, compared with the levels in the Control group, ENO2, MAP2, RBFOX3, and TAU were upregulated in the NOTCH(+) group, while RBFOX3 was downregulated and ENO2 and MAP2 were upregulated in the NOTCH(−) group; there was no statistically significant difference in TAU, but their transcription levels were lower than those in the NOTCH(+) group (Fig. 7D). These results indicated that activation of the NOTCH signaling pathway promoted the differentiation of gADSCs into neurocytes, whereas inhibition of the NOTCH signaling pathway had the opposite effect.

## Discussion

Regenerative medicine is at the forefront of health science [32] with broad application prospects in promoting the regeneration of natural cell lines, growth of new tissues or organs, and disease modeling [33]. Stem cell therapy has attracted considerable attention due to its potentially significant therapeutic effects [34–36]. However, there remain challenges with the potential clinical application of ESCs and iPSCs, including ethical issues, genomic instability, tumorigenicity, and immune rejection reactions [7]. Meanwhile, ADSCs are easily accessible, safety, and have abundant sources, stable properties, and multidirectional differentiation potential, and hence are ideal seed cells for stem cell therapy and regenerative medicine [9, 10]. This study found that miR-204-5p plays an important role in regulating ADSCs differentiation into adipocytes, neurocytes, and hepatocytes; therefore, miRNAs can be used to regulate ADSCs to achieve efficient directional differentiation in vitro and apply them to stem cell therapy and regenerative medicine. The formation of the three germ layers is one of the main events determining cell fate during early embryonic development. The small size and limited number of cells in early embryos hinder related research; in vitro research using stem cells can provide novel insights into early embryo development [37]. Therefore, this study provides insights into early embryonic development by exploring the regulatory mechanisms of ADSCs differentiation into cells derived from the three germ layers.

Adipocytes, the decisive cell type in adipose tissue, are the main site of energy storage and participate in several important physiological processes such as lipid metabolism, systemic energy homeostasis, and systemic insulin sensitivity [38]. Most adipocytes differentiate from MSCs [39]; therefore, it is important to understand the molecular mechanisms underlying ADSCs differentiation into adipocytes. Although adipocyte differentiation is regulated by several factors, such as hormones and cytoskeletal proteins, the most important step is the inhibition or promotion of this process through adipogenesis regulators, including PPARG and C/EBP [38]. PPARG is the main regulatory factor in adipogenesis and plays a decisive role in adipocyte differentiation [40]. This study found that PPARG was downregulated during adipocyte differentiation in the LV_miR group, which may explain why miR-204-5p overexpression inhibited the differentiation of gADSCs into adipocytes. He et al. [27] found that miR-204-5p promoted the differentiation of gADSCs into adipocytes by controlling the expression of DVL3, a key regulator of the Wnt/β-catenin signaling pathway. Conversely, we found that miR-204-5p inhibited the differentiation of gADSCs into adipocytes. This discrepancy may be attributed to the fact that miR-204-5p was transiently transfected in the study of He et al., whereas in this study, we used a lentivirus vector. Another reason is that adipocyte differentiation is highly complex and may vary among different sources and sexes [38]. We found that miR-204-5p activated the AMPK signaling pathway by promoting the expression of ADIPOQ and LEP, inhibiting the expression of PPARG and differentiation of gADSCs into adipocytes. The activation of AMPK during early differentiation can inhibit the expression of PPARG and C/EBPα and further inhibit adipocyte differentiation [41]; our findings support this hypothesis. JAG1, a key protein activating the NOTCH signaling pathway, negatively regulates adipocyte differentiation; overexpression of JAG1 in BMSCs can reduce the expression of PPARG and FABP4, inhibiting the formation of adipocytes [42]. Our study showed that miR-204-5p inhibited gADSCs differentiation into adipocytes by promoting the expression of JAG1 and NOTCH3. It can be seen that JAG1 also negatively regulated the differentiation of gADSCs into adipocytes. Obesity is associated with Type II diabetes, dyslipidemia, hypertension, cardiovascular disease, and cancer [43], and is characterized by an increase in adipocyte volume and number. This study found that miR-204-5p inhibited adipocyte differentiation by acting on the AMPK signaling pathway and the JAG1/NOTCH3 axis, suggesting potential targets for the treatment of obesity and other related metabolic diseases. Insulin gene expression is increased in adipocytes differentiated from ADSCs, which is crucial for the treatment of type I diabetes [44]. The regulatory mechanism identified in this study provides a new method for efficiently differentiating ADSCs into adipocytes for their application in the treatment of Type I diabetes.

With the increase in the global aging population, the incidence of neurodegenerative diseases has increased, yet there remains a lack of effective treatments and drugs in clinical practice; in this context, stem cell therapy is a hopeful approach for treatment [2]. ADSCs have been shown to differentiate into neurocytes in vitro, but their low differentiation efficiency limits their clinical application. Previous studies to overcome this limitation have reported that ghrelin can promote neurocyte differentiation of ADSCs by acting on the AKT/mTOR and β-catenin signaling pathways [45]. Matrigel has a promoting effect on the differentiation of ADSCs into neurocytes [46]. Furthermore, photobiological regulation is a potentially new strategy to induce ADSCs differentiation into neurocytes [47]. Herein, we found that miR-204-5p overexpression can promote gADSCs differentiation into neurocytes. The study acts as a reference for the efficient differentiation of ADSCs into neurocytes and their application in stem cell therapy for neurodegenerative diseases and provides a cell model for drug screening against neurological-related diseases. In addition, we found that the promoting effect of miR-204-5p on the differentiation of gADSCs into neurocytes was achieved via the JAG1/NOTCH3 axis. A previous study has shown that the expression of NOTCH3 promoted neurocyte differentiation [48], which is in line with our findings. miR-204-5p may also promote the differentiation of gADSCs into neurocytes by upregulating the expression of plexin-B2, CEND1, and BRSK1 and downregulating that of VIM. The specific mechanism remains to be determined. In summary, these findings expand our understanding of neurocyte differentiation and provide insights into the involvement of miRNA in this process, which will help to facilitate the development of methods for neural tissue regeneration.

Liver disease has high morbidity and mortality and can lead to “hepatocyte dysfunction” and, ultimately, organ failure [49]. Currently, liver transplantation and cell therapy are two methods for treating end-stage liver disease and liver failure; however, the lack of liver donor organs in liver transplantation and shortage of hepatocytes in cell therapy limit their application [50]. Therefore, it is necessary to identify a new source of cells. ADSCs derived from adipose tissue may be a potential source of cells for hepatocyte therapy [49, 51]. The differentiated hepatocytes of ADSCs have similar biological characteristics and physiological functions as primitive hepatocytes, including the expression of specific genes of AFP and various cytochrome P450 (CYP450) subtypes as well as the secretion of ALB and urea [52, 53]. We found that, compared with uninduced cells, the differentiated hepatocytes from ADSCs contained glycogen vesicles and secreted ALB and urea. The marker genes AFP, ALB, HNF4A, and KRT18 were highly expressed, indicating that gADSCs can induce differentiation into hepatocytes in vitro. Although ADSCs can differentiate into hepatocytes, efficient in vitro induction of differentiation remains challenging. Herein, the overexpression of miR-204-5p promoted gADSCs differentiation into hepatocytes, which demonstrates the potential application of cell therapy for liver diseases. Primitive hepatocytes can be used for drug screening, yet are difficult to maintain in an in vitro culture [54]. In this study, hepatocytes differentiated from gADSCs overexpressing miR-204-5p were maintained for an extended period in vitro, providing a new approach for constructing a cell model for drug screening in liver diseases. We also found that only E2F8 was associated with hepatocyte differentiation among the differentially expressed genes and proteins associated with hepatocytes. E2F8 plays an essential role in embryonic development and regulation of cell function and can induce polyploidization of hepatocytes by inhibiting CDK1, which effects cell differentiation, cycle, proliferation, and apoptosis [55]. Herein, E2F8 was upregulated in the LV_miR group, suggesting the promoting effect of miR-204-5p on gADSCs differentiation into hepatocytes is mediated by an increase in E2F8 expression.

Overall, our study found that gADSCs can differentiate into cells derived from three different germ layers in vitro: adipocytes, neurocytes, and hepatocytes, and detailed the molecular mechanism underlying miR-204-5p regulation of gADSCs differentiation into cells from the three germ layers. These results provide novel insights into the regulatory mechanisms of early embryonic development and help to facilitate ADSCs application in stem cell therapy and regenerative medicine.

## Materials and methods

### Materials

Primary Arbas Cashmere goat ADSCs were isolated, identified and stored in our laboratory as previously described [56]. The lentiviral vector GV369 and virus packaging helper plasmids Helper 1.0 and Helper 2.0 were purchased from Shanghai Genechem Co., Ltd (Shanghai, China). mirVana™ miRNA Isolation Kit, BCA protein quantitative analysis kit, and Pierce ECL Western Blotting Substrate kit were purchased from Thermo Fisher Scientific (Waltham, USA). TaqMan® MicroRNA Reverse Transcription Kit and TaqMan® Universal Master Mix II, no UNG were purchased from Applied Biosystems (Carlsbad, USA). DMEM/F12, IMDM, PBS, PS, Fetal bovine serum (FBS), and Trypsin EDTA were purchased from Gibco (Grand Island, USA). DMSO, Dexamethasone, Biotin, Insulin, Pantothenate, IBMX, Rosiglitazone, Oil Red O, Nicotinamide, ITS + 1 liquid medium supplement, BSA, Tween 20, APS, and TEMED were purchased from Sigma-Aldrich (Saint Louis, USA). Standard rabbit serum, EGF, and bFGF were purchased from Invitrogen (Carlsbad, USA). β-mercaptoethanol and glycogen PAS staining kits were purchased from SolarBio (Beijing, China). ENO2 (Cat# 10149-1-AP, RRID: AB_2099180), TAU (Cat# 10274-1-AP, RRID: AB_2139718), MAP2 (Cat# 17490-1-AP, RRID: AB_2137880), RBFOX3 (Cat# 23060-1-AP, RRID: AB_11232408), AFP (Cat# 14550-1-AP, RRID: AB_2223933), ALB (Cat# 16475-1-AP, RRID: AB_2242567), Cytokeratin 18 (Cat# 10830-1-AP, RRID: AB_2133164), alpha-tubulin (Cat# 11224-1-AP, RRID: AB_2210206), Adiponectin (Cat# 21613-1-AP, RRID: AB_2878891), Leptin (Cat# 17436-1-AP, RRID: AB_2265702), AMPK Alpha 1 (Cat# 10929-2-AP, RRID: AB_2169568), PPAR gamma (Cat# 16643-1-AP, RRID: AB_10596794), NOTCH3 (Cat# 55114-1-AP, RRID: AB_10858393), Plexin-B2 (Cat# 10602-1-AP, RRID: AB_2166424), BRSK1 (Cat# 12673-1-AP, RRID: AB_2274933), CEND1 (Cat# 13280-1-AP, RRID: AB_2291632), vimentin (Cat# 10366-1-AP, RRID: AB_2273020), E2F8 (Cat# 13425-1-AP, RRID: AB_2097273) rabbit polyclonal antibodies, and Jagged1 (Cat# 66890-1-Ig, RRID: AB_2882220) mouse monoclonal antibody were purchased from Proteintech (Wuhan, China). Anti-HNF-4-alpha antibody (Cat# ab92378, RRID: AB_10562973), Goat Anti-Rabbit IgG H&L (HRP) (Cat# ab6721, RRID: AB_955447), Goat Anti-Rabbit IgG H&L (FITC) (Cat# ab6717, RRID: AB_955238), and Goat Anti-Rabbit IgG H&L (Alexa Fluor® 647) (Cat# ab150079, RRID: AB_2722623) were purchased from Abcam (Cambridge, UK). Goat nerve growth factor (NGF) ELISA kit and albumin (ALB) ELISA kit were purchased from Wuhan Enlibio Biotech Co., Ltd (Wuhan, China). HGF and OSM were purchased from Peprotech. QuantiChrom^TM^ Urea Assay Kit was purchased from Bioassy Systems (Hayward, USA). RNAiso, PrimeScript^TM^ RT Reagent Kit with gDNA Eraser, and SYBR® Premix Ex Taq^TM^ II (Tli RNaseH Plus) were purchased from Takara (Kyoto, Japan). The mammalian protein extraction reagents, SDS-PAGE electrophoretic solution, and transfer membrane solutions were purchased from CwBiotech (Beijing, China). AICAR (Acadesine), dorsomorphin (Compound C) 2HCl, LY411575, and Valproic acid (VPA) were purchased from Selleck (Houston, USA). The cell culture dishes were purchased from Corning (Corning, USA).

### Methods

#### Cell culture

gADSCs were removed from the liquid nitrogen, thawed rapidly in a water bath at 37 °C, and cultured in DMEM/F12 containing 15% FBS in an incubator at 37 °C with 5% CO_2_. The culture medium was first changed on the next day, then every two days. When the confluence of adherent cells reached 80–90%, cells were trypsinized with 0.25% trypsin/EDTA for 3 min for passage culture.

#### Infection of gADSCs with lentiviral vector

Cells were seeded in 6-well plates at 3–5×10^4^ cells/mL. The following day, the lentiviral vector was transfected into gADSCs according to the infection conditions determined pre-experimentally (MOI = 20, infection enhancement medium ENi.S. + Polybrene). After 48 h of infection, the cells were transferred from the 6-well plates into 100 mm culture dishes and cultured for 24 h, following which puromycin at a final concentration of 2 mg/mlL was added for screening and maintained for 14 days. Selected cells were digested, cultured, and frozen.

#### Detection of infection efficiency of lentiviral vector

Green fluorescence intensity of gADSCs infected with the lentiviral vector was observed under a fluorescence microscope to determine the infection efficiency of the lentiviral vector. The infection efficiency of the lentiviral vector was further determined using flow cytometric analysis of the proportion of cells with green fluorescence among the total cells. Finally, the expression of miR-204-5p was detected using real-time qPCR as follows: Cells were collected in enzyme-free tubes, and miRNA was extracted according to the instructions of mirVana™ miRNA Isolation Kit. Reverse transcription of miRNAs was performed according to the instructions of TaqMan® MicroRNA Reverse Transcription Kit. The transcription of miR-204-5p in gADSCs was detected using miRNA real-time qPCR according to the instructions of TaqMan® Universal Master Mix II, no UNG, using U6 snRNA as the internal reference and using 2^-ΔΔCt^ for analysis.

#### Induction of gADSCs differentiation into adipocytes

Differentiation of uninfected gADSCs (control group), gADSCs infected with the empty lentiviral vector (LV_Con group), and gADSCs infected with miR-204-5p in the lentiviral vector (LV_miR group) into adipocytes was induced simultaneously. The culture medium was discarded, cells were washed with PBS, and cultured with the adipocyte-inducing differentiation medium (DMEM/F12 + 3% FBS + 1% PS + 1 μmol/L Dex + 33 μmol/L Bio + 17 μmol/L Pan + 1 μmol/L Insulin + 0.5 mmol/L IBMX + 5 μmol/L Rosiglitazone + 5% Rabbit Serum) for 3 days and replaced with maintenance medium devoid of IBMX and rosiglitazone; the medium was changed every 3 days [57].

#### Oil red O staining and quantitative detection of lipid droplets

After adipogenic induction, cells were stained with 0.5% Oil Red O and observed to identify adipocytes after 20 min. After staining and identification, Oil Red O was discarded and air dried; isopropanol was added to dissolve the lipid droplets, absorbance of 550 nm was detected, and the lipid droplet content of the adipocytes was determined.

#### Transcription of adipocyte marker genes was detected using real-time qPCR

Total RNA was extracted using RNAiso, and reverse transcription was performed according to the instructions of the PrimeScript^TM^ RT Reagent Kit with gDNA Eraser. The transcription of the adipocyte marker genes PPARG, ADIPOQ, PERILIPIN, LEP, and IRS1 was detected using real-time qPCR according to the instructions of SYBR® Premix Ex Taq^TM^ II (Tli RNaseH Plus), GAPDH was used as the internal reference. The primer sequences are listed in Table S1.

#### Induction of gADSCs differentiation into neurocytes

Differentiation of Control, LV_Con, and LV_miR groups into neurocytes was induced. The culture medium was discarded, cells were washed with PBS, cultured with the neurocyte pre-induction medium (DMEM/F12 + 10 ng/mL EGF + 10 ng/mL bFGF) for 2 days, and replaced with the neurocyte differentiation medium (DMEM/F12 + 10 mM BME) for 8 h [57].

#### Detection of NGF secreted by neurocytes using ELISA

The culture supernatants of neurocytes differentiated from the Control, LV_Con, and LV_miR groups were collected. NGF content in the cell supernatant was detected according to the instructions of the goat nerve growth factor (NGF) ELISA kit.

#### Cellular immunofluorescence for expression analysis of neurocyte marker genes

The expression of the neurocyte marker genes ENO2, TAU, MAP2, and RBFOX3 was identified using cellular immunofluorescence. Cells were seeded in 24-well plates, grown to > 80% confluence, fixed with 4% paraformaldehyde for 15 min, permeabilized with 0.5% TritonX-100 for 10 min, blocked in 1% BSA for 1 h, and incubated with primary antibody (1:300) overnight at 4 °C. The next day, the liquid was discarded, and cells were washed three times with PBS, incubated with Goat Anti-Rabbit IgG H&L (Alexa Fluor ® 647) (1:300) for 1 h at room temperature, and stained with DAPI for 5 min. The expression of target proteins in the cells was observed using confocal microscopy.

#### Transcription detection of neurocyte marker genes using real-time qPCR

Transcription of the neurocyte marker genes ENO2, TAU, MAP2, and RBFOX was detected using real-time qPCR. GAPDH was used as an internal reference; the primer sequences are listed in Table S1.

#### Induction of gADSCs differentiation into hepatocytes

Differentiation of the Control, LV_Con, and LV_miR groups into hepatocytes was induced simultaneously. The culture medium was discarded, and cells were washed with PBS, cultured with the hepatocyte preinduction medium (IMDM + 20 ng/mL EGF + 10 ng/mL bFGF) for 2 days, and replaced with the hepatocyte differentiation medium (IMDM + 20 ng/mL HGF + 10 ng/mL bFGF+0.61 g/L Nicotinamide) for 3 days. The medium was replaced with the hepatocyte maturation medium (IMDM + 20 ng/mL OSM + 1 μmol/L Dexamethasone + 50 ng/mL ITS + 1 Liquid Media Supplement) for an additional 3 days to complete the stage of maturation culture.

#### Identification of hepatocytes using glycogen PAS staining

Periodic acid-Schiff (PAS) staining was used to identify the differentiated hepatocytes by observing the distribution of glycogen vesicles. The culture medium was discarded, and cells were washed with PBS and fixed with PAS stationary liquid for 15 min at room temperature. The cells were washed twice, dried, oxidized at room temperature for 20 min, oxidized twice, stained with Schiff’s reagent at room temperature for 30 min, washed twice with sodium sulfite solution and distilled water, and stained with Mayer’s hematoxylin staining solution for 5 min before microscopic examination.

#### Detection of ALB and urea content

The culture supernatants of hepatocytes differentiated from the Control, LV_Con, and LV_miR groups were collected. The ALB and urea contents in the cell supernatant were detected according to the instructions of the goat albumin (ALB) ELISA kit and QuantiChrom^TM^ Urea Assay kit.

#### Cellular immunofluorescence for expression analysis of hepatocyte marker genes

The expression of the hepatocyte marker genes AFP, ALB, HNF4A, and KRT 18 in the Control, LV_Con, and LV_miR groups was determined using cellular immunofluorescence, using a similar method described for the expression of neurocyte marker genes.

#### Transcription detection of hepatocyte marker genes using real-time qPCR

Transcription of the hepatocyte marker genes AFP, ALB, HNF4A, and KRT18 was detected using real-time qPCR. GAPDH was used as an internal reference; the primer sequences are listed in Table S1.

#### RNA-Seq

RNA-Seq was performed on the Control, LV_Con, and LV_miR group before differentiation. Total RNA was extracted from the control, LV_Con, and LV_miR groups. RNA quality was evaluated via electrophoresis using an Agilent 2100 Bioanalyzer (Agilent Technologies, San Diego, CA, USA). Sequencing of the libraries was performed on an Illumina NovaSeq 6000 instrument by Shanghai Majorbio Bio-pharm Technology Co., Ltd. (Shanghai, China) and individually assessed for quality using FastQC. Quality control data were compared with the reference genome (Capra_hircus, http://asia.ensembl.org/Capra_hircus/Info/Index) using HISAT2 (http://ccb.jhu.edu/software/hisat2/index.shtml) to obtain mapped reads for subsequent analyses. Differential expression analysis was performed using DESeq2 (http://bioconductor.org/packages/stats/bioc/DESeq2) with *P*-adjust < 0.05 & |log2FC|≥ 0.585. Gene Ontology and Kyoto Encyclopedia of Genes and Genomes pathway analyses were performed using KOBAS2.1.1 (http://kobas.cbi.pku. edu.cn/).

#### Validation of RNA-Seq results using real-time qPCR

Real-time qPCR was used to detect the transcription of differentially expressed genes in the Control, LV_Con, and LV_miR groups to verify the reliability of the RNA-Seq results. GAPDH was used as the internal reference gene, and 2^-ΔΔCt^ was used to analyze the transcriptional of differential genes. The primer sequences are listed in Table S1.

#### TMT-labeled quantitative proteomics

TMT-labeled quantitative proteomics was performed on the Control, LV_Con, and LV_miR group cells before differentiation. Proteins from the Control, LV_Con, and LV_miR groups were extracted using the mammalian protein extraction reagents. Protein concentrations were determined according to the instructions of the Pierce BCA kit (Thermo Scientific); the extracted proteins were identified using sodium dodecyl-sulfate polyacrylamide gel electrophoresis (SDS-PAGE). Sequencing of the mass spectrometry was performed by Shanghai Majorbio Bio-pharm Technology Co., Ltd. (Shanghai, China). The raw mass spectrometry data were matched with the protein sequence of goats (Capra_hircus) in the NCBI database using Proteome Discoverer to obtain protein-related information. The obtained proteins were subjected to statistical analysis and quality control using the online platform Majorbio Cloud Platform (www.majorbio.com). After quality control, the proteins were compared with databases such as GO, KEGG, COG, and Pfam to obtain protein annotation information. The screening criteria for differentially expressed proteins were FC > 1.2 or <0.83, *P* < 0.05. GO and KEGG enrichment analyses of the differential proteins were performed using BLAST2GO2.5.0 and KOBAS2.1.1.

#### Proteomics results validation using western blotting

The expression of the differential proteins in the Control, LV_Con, and LV_miR groups was detected using western blotting, with α-Tublin as the internal reference. Protein samples were separated using SDS-PAGE (90 V/30 min, 120 V/90 min) and transferred to 0.22 μm nitrocellulose membranes. The membranes were blocked with 5% skim milk powder for 1 h at room temperature, and incubated with the primary antibody (1:1,000) overnight at 4 °C. The following day, the membranes were washed thrice with TBST and incubated with the secondary antibody Goat Anti-Rabbit IgG H&L (HRP) (1:10,000) for 1 h at room temperature. Finally, protein bands were visualized using the Pierce ECL Western Blotting Substrate kit, and their intensities were quantified using Tanon-5200. Gray-scale analysis was performed using the ImageJ software.

#### gADSCs treated with pathway activators and inhibitors to induce differentiation into adipocytes

gADSCs were seeded and cultured in 6-well plates. When the cell confluence reached 50%, cells were treated with 0.2 mM AICAR (AMPK activator), 4.0 μM Dorsomorphin (Compound C) 2HCl (AMPK inhibitor), 0.4 mM Valproic acid (VPA) (NOTCH activator), and 1.2 μM LY411575 (NOTCH inhibitor) for 24 h. Untreated gADSCs and gADSCs treated with AICAR, Dorsomorphin (Compound C) 2HCl, Valproic acid (VPA), and LY411575 were simultaneously differentiated into adipocytes. The induction and identification were conducted using a method similar to that of the induction of gADSCs differentiation into adipocytes in this study.

#### Treatment of gADSCs with pathway activators and inhibitors to induce differentiation into neurocytes

gADSCs were seeded and cultured in six-well plates. When the cell confluence reached 70%, cells were treated with 0.4 mM valproic acid (VPA) and 1.2 μM LY411575 for 24 h. Untreated gADSCs and gADSCs were simultaneously treated with valproic acid (VPA) and LY411575 differentiated into neurocytes. The induction and identification were conducted using a method similar to that of the induction of gADSCs differentiation into neurocytes in this study.

#### Statistical analysis of results

Data were analyzed using SPSS 19.0 software (IBM, Chicago, IL, USA) and are presented as mean ± standard deviation (SD). The differences were compared using Student’s *t* test, and the *P* value was determined using the SPSS 19.0 software. *P* < 0.05 was considered statistically significant. All experiments were independently repeated three times.

### Supplementary information

Figure S1

Supplementary figure legends

Table S1

Original western blots

## Data Availability

Supplementary information is available on Cell Death Discovery’s website. The transcriptomics data generated in this publication have been deposited to the NCBI Sequence Read Archive (SRA) (https://www.ncbi.nlm.nih.gov/sra) and are accessible through SRA accession number PRJNA987813. The mass spectrometry proteomics data generated in this publication have been deposited to the ProteomeXchange Consortium (http://proteomecentral.proteomexchange.org) via the iProX partner repository with the dataset identifier PXD043379.
