# Supplementary figures and images for: Molecular mechanism underlying miR-204-5p regulation of adipose-derived stem cells differentiation into cells from three germ layers
Source: Cell Death Discov. 2024 Feb 22;10:95. doi: 10.1038/s41420-024-01852-4 (PMC10884001; doi:10.1038/s41420-024-01852-4)

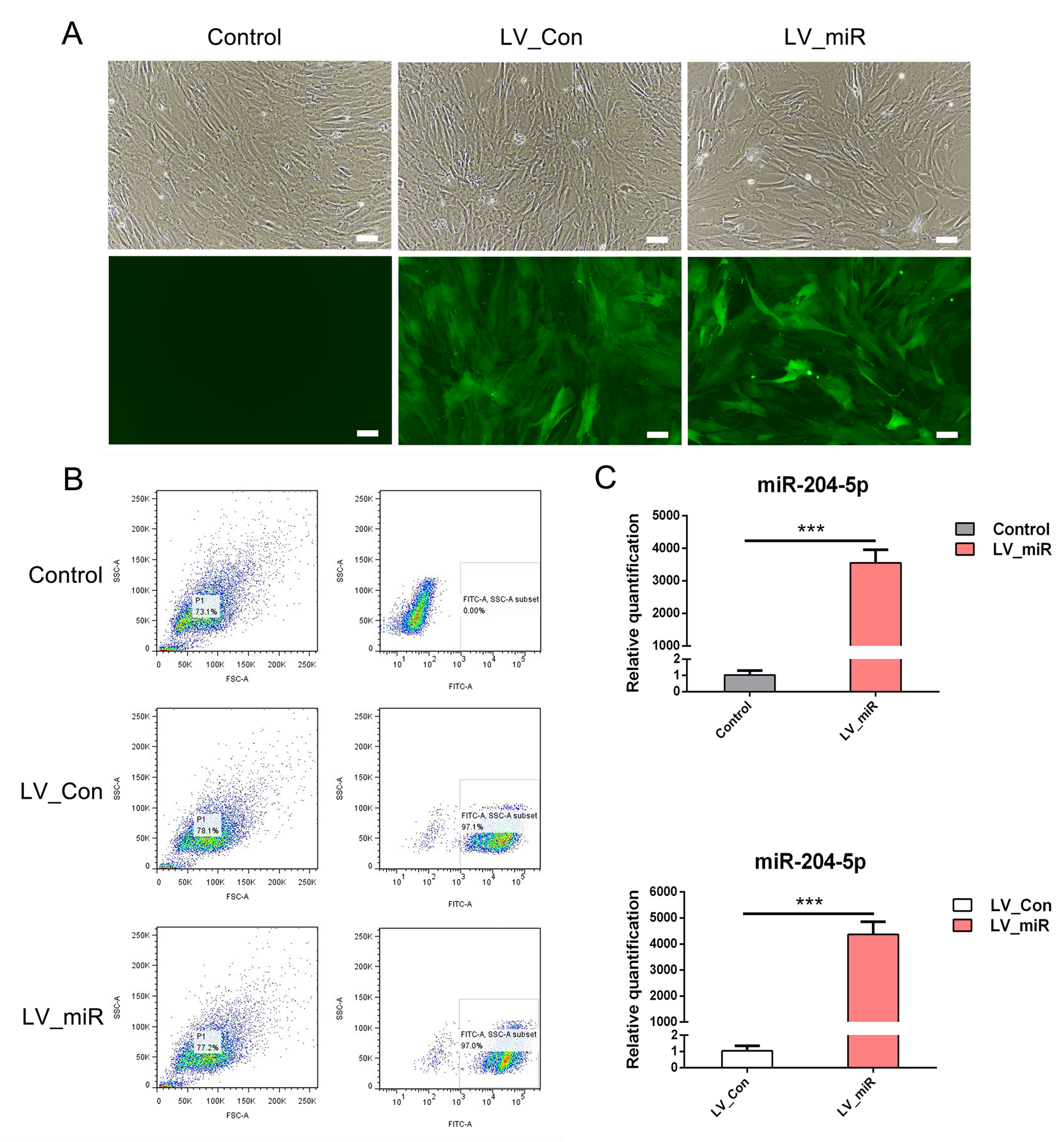

Supplement: Supplementary file 1 — Figure S1 [file 41420_2024_1852_MOESM1_ESM.tif]
