# Supplementary material for: Molecular mechanism underlying miR-204-5p regulation of adipose-derived stem cells differentiation into cells from three germ layers
Source: Cell Death Discov. 2024 Feb 22;10:95. doi: 10.1038/s41420-024-01852-4 (PMC10884001; doi:10.1038/s41420-024-01852-4)
Supplement: Supplementary file 2 — Supplementary figure legends [file 41420_2024_1852_MOESM2_ESM.doc]

**Supplementary figure legends**

**Fig. S1 Detection of infection efficiency of miR-204-5p up lentiviral vector.** **A** Infection efficiency of the lentiviral vector observed using a fluorescence microscope (Bar: 100 μm). **B** Infection efficiency of the lentiviral vector detected using flow cytometry. **C** Infection efficiency of the lentiviral vector and the transcription of miR-204-5p detected using real-time qPCR. Data were presented as mean ± SD; P values were determined by a two-tailed unpaired t test, ***P < 0.01.
