# Supplementary material for: Molecular mechanism underlying miR-204-5p regulation of adipose-derived stem cells differentiation into cells from three germ layers
Source: Cell Death Discov. 2024 Feb 22;10:95. doi: 10.1038/s41420-024-01852-4 (PMC10884001; doi:10.1038/s41420-024-01852-4)
Supplement: Supplementary file 3 — Table S1 [file 41420_2024_1852_MOESM3_ESM.doc]

**Supplementary table 1** Primer sequences used for real-time quantitative PCR

| Gene Name |  | Sequence | Product length (bp) |
| --- | --- | --- | --- |
| GAPDH | F | 5′- TTCCACGGCACAGTCAAGG -3′ | 114 |
|  | R | 5′- CTCAGCACCAGCATCACCC -3′ |  |
| PPARG | F | 5′- CAAGAGTACCAAAGTGCAATCAA -3′ | 144 |
|  | R | 5′- TGACGCTTTATCCCCACAGA -3′ |  |
| ADIPOQ | F | 5′- GGTGAGAAGGGTGAGAAAGGAG -3′ | 209 |
|  | R | 5′- TGGTAAAGCGAATGGGAACA -3′ |  |
| PERILIPIN | F | 5′- TGGACCACTTGGTGGAGTATGT -3′ | 104 |
|  | R | 5′- CTATTTCTTCTTTTCTGGGGCTTTC -3′ |  |
| LEPTIN | F | 5′- AGGTGGGAAATGTGTTGATGG -3′ | 115 |
|  | R | 5′- TGAAAACCGCAAAGATGAGAAG -3′ |  |
| IRS1 | F | 5′- CTACCGGCACTCTGCCTTC -3′ | 139 |
|  | R | 5′- GGACATGGGCATGTAGCCA -3′ |  |
| ENO2 | F | 5′- TGGAACTGGAAGGGTAGCAGA -3′ | 95 |
|  | R | 5′- GGGAGAGGGACAGGAGACAA -3′ |  |
| TAU | F | 5′- GTCTCGTGTCTGGCTTTCCA -3′ | 140 |
|  | R | 5′- GGTTACAAGCCAGCATCCCT -3′ |  |
| MAP2 | F | 5′- TGAAATCCCACCCACAAGCA -3′ | 160 |
|  | R | 5′-TGCCTGATGACCCCATTTCC -3′ |  |
| RBFOX3 | F | 5′- ACCAACGGCTGGAAGCTAAA -3′ | 159 |
|  | R | 5′- GCAGCCCGGAACGTATTGTA -3′ |  |
| AFP | F | 5′- AAAATTTGGACCCCGGACCT -3′ | 149 |
|  | R | 5′- GACACTCCAGCACGTTTCCT-3′ |  |
| ALB | F | 5′- AACCTATGGTGACATGGCCG-3′ | 139 |
|  | R | 5′- TGCCTTAAACTCGGCACACA -3′ |  |
| HNF4A | F | 5′- TTTGACCCAGATGCCAAGGG-3′ | 105 |
|  | R | 5′- GTCATACTGGCGGTCGTTGA -3′ |  |
| KRT18 | F | 5′- TTGCGGACCTCACACACTAC-3′ | 104 |
|  | R | 5′- TTGGAGAAGGTGGATTGGGC -3′ |  |
| AMPK | F | 5′-GCGCCATACCCTCGATGAAT-3′ | 191 |
|  | R | 5′- TCTTCCTCCGAACACGCAAA -3′ |  |
| JAG1 | F | 5′- AAACGGGTGGAAAGGCAAGA-3′ | 109 |
|  | R | 5′-CATGCACTTGAAAGCGTCCC-3′ |  |
| NOTCH3 | F | 5′- ACTAACCCAGTGAATGGCCG-3′ | 147 |
|  | R | 5′-CAGGAATGAGCCCTGTGTGT-3′ |  |
| plexin-B2 | F | 5′-GCTGTGCCTCTTTGGAGGAT-3′ | 182 |
|  | R | 5′-AGGGATACTGGTGGGAGGTC-3′ |  |
| CEND1 | F | 5′-TCTTGAGAACTTGACCCCGC-3′ | 166 |
|  | R | 5′- GAATTCGGCATGCACTGGAC -3′ |  |
| BRSK1 | F | 5′-GTTCTTCCGCCAGATCGTGT-3′ | 116 |
|  | R | 5′- CTGCGATGCGGATGTTGTTT -3′ |  |
| VIM | F | 5′-TGACCTGGAGCGTAAAGTGG-3′ | 185 |
|  | R | 5′- CCACGCTCTCATACTGCTGA -3′ |  |
| E2F8 | F | 5′-CCATCCTGAGCCAGAACCAA-3′ | 169 |
|  | R | 5′- GCACCATCAAAATCCACGCA -3′ |  |
