# Supplementary material for: Molecular mechanism underlying miR-204-5p regulation of adipose-derived stem cells differentiation into cells from three germ layers
Source: Cell Death Discov. 2024 Feb 22;10:95. doi: 10.1038/s41420-024-01852-4 (PMC10884001; doi:10.1038/s41420-024-01852-4)

Original western blots

Fig.3J_α-Tublin

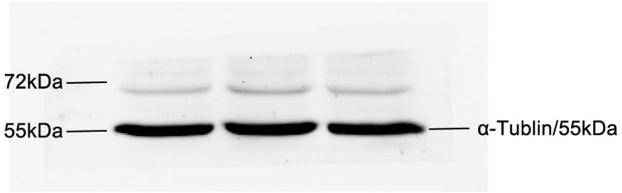

Fig.3J_ADIPOQ

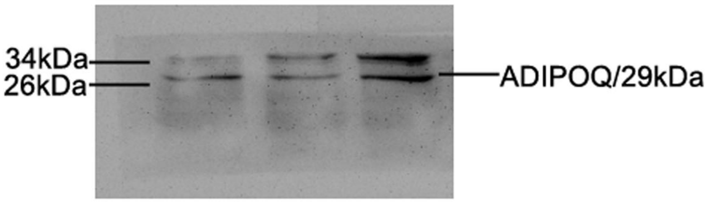

Fig.3J_AMPK

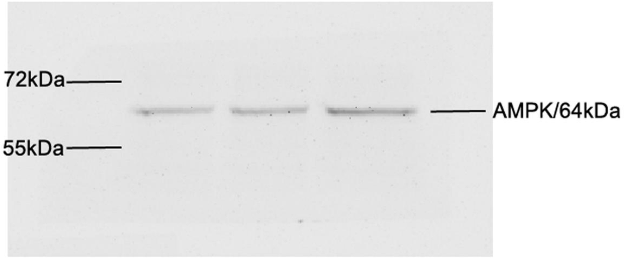

Fig.3J_PPARG

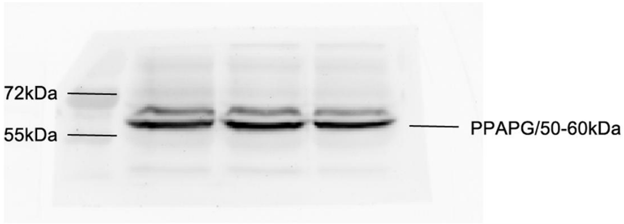

Fig.3J_JAG1

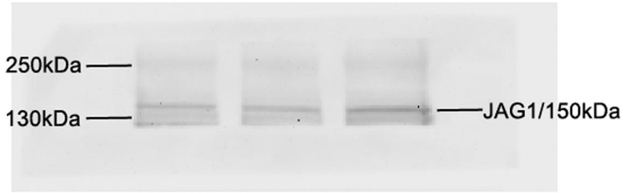

Fig.3J_NOTCH3

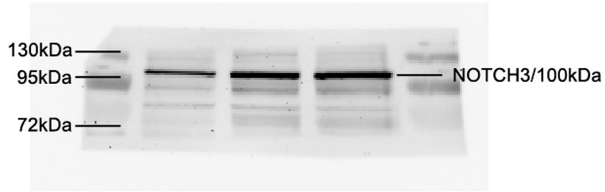

Fig.4J_α-Tublin

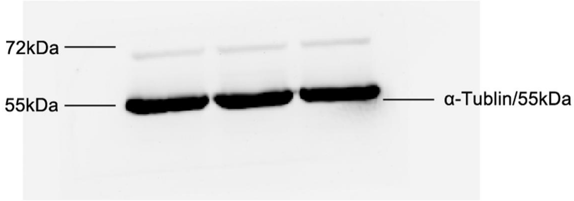

Fig.4J_plexin-B2

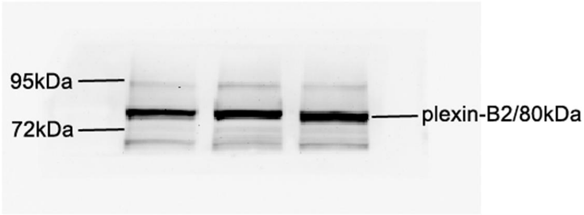

Fig.4J_VIM

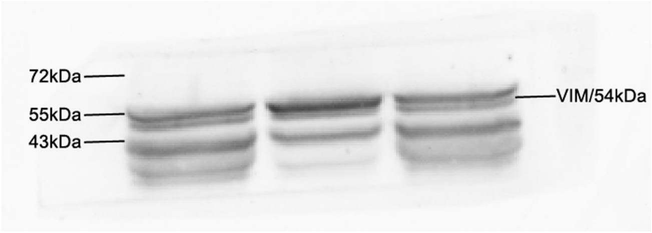

Fig.5I_α-Tublin

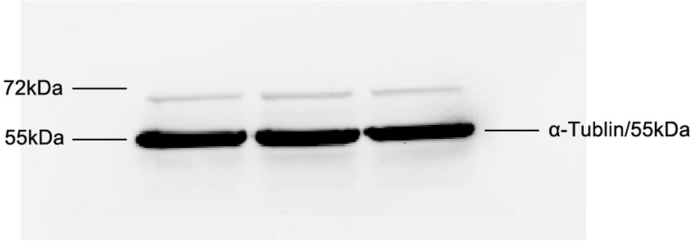

Fig.5I_E2F8

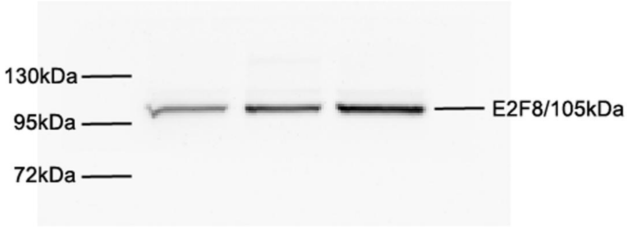

Fig.6B_α-Tublin

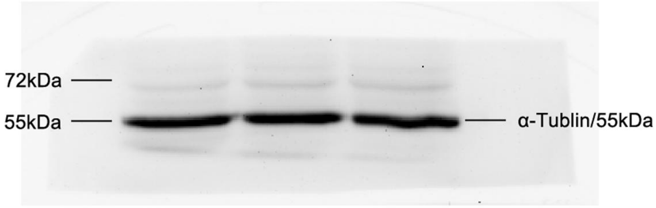

Fig.6B_ADIPOQ

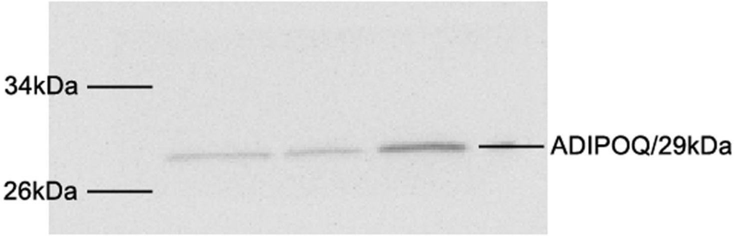

Fig.6B_AMPK

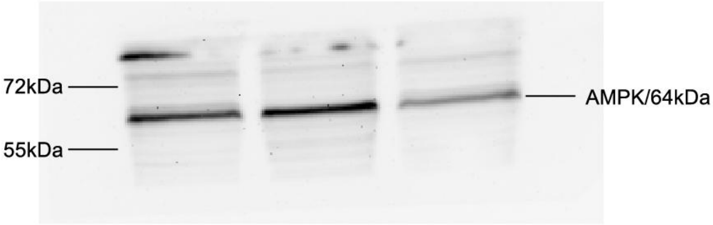

Fig.6B_PPARG

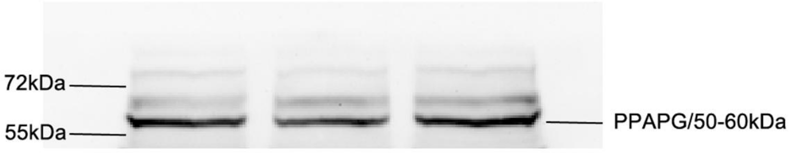

Fig.6D_α-Tublin

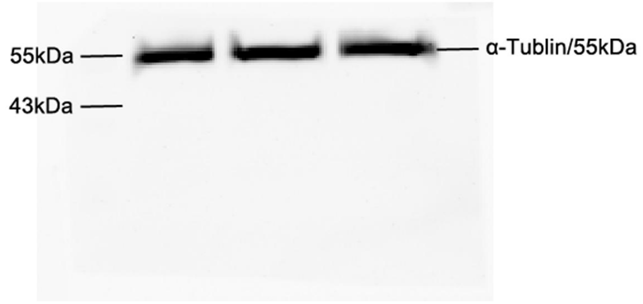

Fig.6D_JAG1

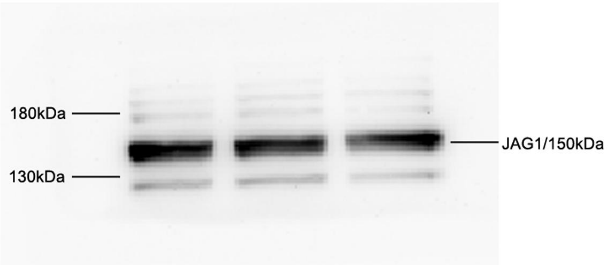

Fig.6D_NOTCH3

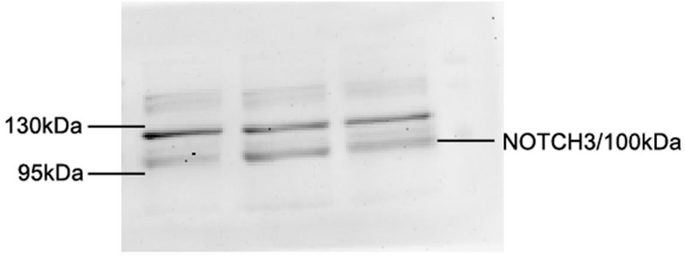

Supplement: Supplementary file 4 — Original western blots [file 41420_2024_1852_MOESM4_ESM.pdf]
